# Kidney function in acromegaly: evidence from a long-term observational study

**DOI:** 10.1007/s11102-025-01520-5

**Published:** 2025-05-06

**Authors:** Giona Castagna, Silvia Ippolito, Sara Cassibba, Liana Cortesi, Emanuele Costi, Ahmad Harb, Luigi Alberto Lanterna, Angelo Mirco Sicignano, Roberto Trevisan, Alessandro Rossini

**Affiliations:** 1 Endocrinology and Diabetes Unit, ASST Papa Giovanni XXIII, Bergamo, Italy; 2 Department of Medicine and Surgery, University of Milano Bicocca, Milan, Italy; 3 Department of Neurosurgery, ASST Papa Giovanni XXIII, Bergamo, Italy

**Keywords:** Acromegaly, Kidney, CKD, IGF-1, eGFR, GH

## Abstract

**Purpose:**

Growth hormone (GH) and insulin-like growth factor 1 (IGF-1) are critical regulators of renal development and function. Acromegaly, characterized by chronic GH hypersecretion, leads to renal hypertrophy and glomerular hyperfiltration. While immediate treatment of acromegaly mitigates hyperfiltration, the long-term risk of renal damage in treated patients remains unclear. Our study aimed to assess renal function over time in patients with acromegaly who were followed long-term at our institution.

**Methods:**

This study analyzed 80 patients with acromegaly from a single center. Creatinine values were recorded to assess kidney function before and after treatment. The estimated glomerular filtration rate (eGFR) was calculated using the CKD-EPI 2021 formula. eGFR variations were evaluated over the first 12 months after treatment (acute slope) and during long-term follow-up with a mean duration of 11.28 years (chronic slope). Descriptive statistics and multivariable regression analyses were performed.

**Results:**

Among the 80 patients (43.7 years, 46 male), 51 underwent surgery (11 of whom also received subsequent radiotherapy), while 29 received exclusively medical therapy. Comorbidities included diabetes (31.25%) and hypertension (65%). eGFR decreased acutely after treatment in all groups, with a more pronounced decline in surgically treated patients (mean − 15.15 mL/min/1.73 m²; *p* = 0.042). The mean chronic eGFR loss was − 1.28 mL/year, with age (OR 1.09 per year) and diabetes (OR 5.66) significantly associated with a greater decline in eGFR (*p* < 0.01).

**Conclusions:**

Renal hyperfiltration in acromegaly tends to normalize following treatment, with a more rapid response observed in patients who undergo surgery. Chronic kidney disease is highly prevalent in acromegaly and is closely linked to diabetes, which further contributes to the increased cardiovascular risk seen in these individuals.

**Supplementary Information:**

The online version contains supplementary material available at 10.1007/s11102-025-01520-5.

## Introduction

Acromegaly is characterized by the hypersecretion of growth hormone (GH), usually caused by a pituitary adenoma, leading to abnormalities in multiple target organs, including the kidney. Exposure to supraphysiological GH levels in patients with acromegaly has been associated with renal hypertrophy, increased glomerular filtration, extracellular volume expansion, and hypercalciuria [1].

Although treatment for acromegaly can improve kidney hyperfiltration [2, 3], no study to date evaluated whether different treatment modalities influence this outcome. Emerging evidence suggests an association between acromegaly and an increased risk of renal cysts [4] and end-stage renal disease (ESRD) [5], yet long-term renal function in this condition remains poorly characterized.

The aim of our study was to evaluate post-treatment renal function and its changes over time in a large cohort of patients followed long-term at our institution.

## Population and methods

Eighty patients with acromegaly from Papa Giovanni XXIII Hospital, Bergamo, were identified through electronic health records. Clinical data were collected from the initial evaluation to the most recent visit. All patients provided informed consent for the anonymized use of their data.

Creatinine values were recorded from the first therapeutic intervention. If pre-treatment values were unavailable, the earliest available creatinine measurement was used. The estimated glomerular filtration rate (eGFR) was calculated using the CKD-EPI 2021 formula. Acute eGFR changes (acute slope) were determined by comparing pre-treatment values with those obtained within 12 months post-treatment. Chronic eGFR decline (chronic slope) was assessed through regression analysis of eGFR values in patients with at least three post-intervention creatinine measurements.

Chronic Kidney Disease (CKD) was defined as an eGFR of less than 60 mL/min/1.73 m², while ESRD as an eGFR of less than 15 mL/min/1.73 m².

Descriptive statistics were used to summarize patient characteristics, with continuous variables presented as mean values (standard deviation) and categorical variables as frequencies and percentages. Multivariable regression analysis was performed to examine acute and chronic slopes across treatment groups, adjusting for relevant covariates. A two-sided p-value ≤ 0.05 was considered statistically significant. All analyses were conducted using STATA 16.1.

## Results

Demographic and clinical characteristics are detailed in Table 1. The mean age at diagnosis was 43.7 (SD 14) years, mean disease duration was 22.3 (SD 12.7) years. Forty-six patients (57.5%) were male. Eight patients (10%) died during follow-up.

Transsphenoidal (TNS) surgery was performed in 51 patients (63.8%), three of whom required a second TNS surgery. Eleven patients (13.7%) underwent radiotherapy, including 9 after the first surgery and 2 after the second. Thirty-one patients (60.8%) did not achieve remission (defined as normalized IGF-1 [6]) and were subsequently treated with medical therapy. Primary medical therapy was administered to 29 patients (36.2%). Overall, 55 out of 80 patients (68.7%) achieved remission. Comorbidities included diabetes in 25 patients (31.3%), and hypertension in 52 (65%).

Among the 80 patients, 29 (36.2%) had pre- and post-treatment creatinine measurements within 12 months of intervention. These patients were categorized into three groups: (i) medical therapy only (*n* = 6), (ii) medical therapy followed by surgery (*n* = 12), and (iii) surgery only (*n* = 11). The mean acute eGFR loss was − 6.64 mL/min/1.73 m² (SD 9.06) in group (i), -3.22 mL/min/1.73 m² (SD 8.56) in group (ii), and − 15.15 mL/min/1.73 m² (SD 13.87) in group (iii), with significant differences between groups (*p* = 0.042, Kruskal-Wallis test).

Multivariable linear regression (Table 2A) examined the relationship between acute eGFR changes and treatment groups, adjusting for demographic and clinical factors. The surgery-only group (iii) exhibited a significantly steeper decline in eGFR compared to the medical therapy group (*p* = 0.049).

**Fig. 1:**
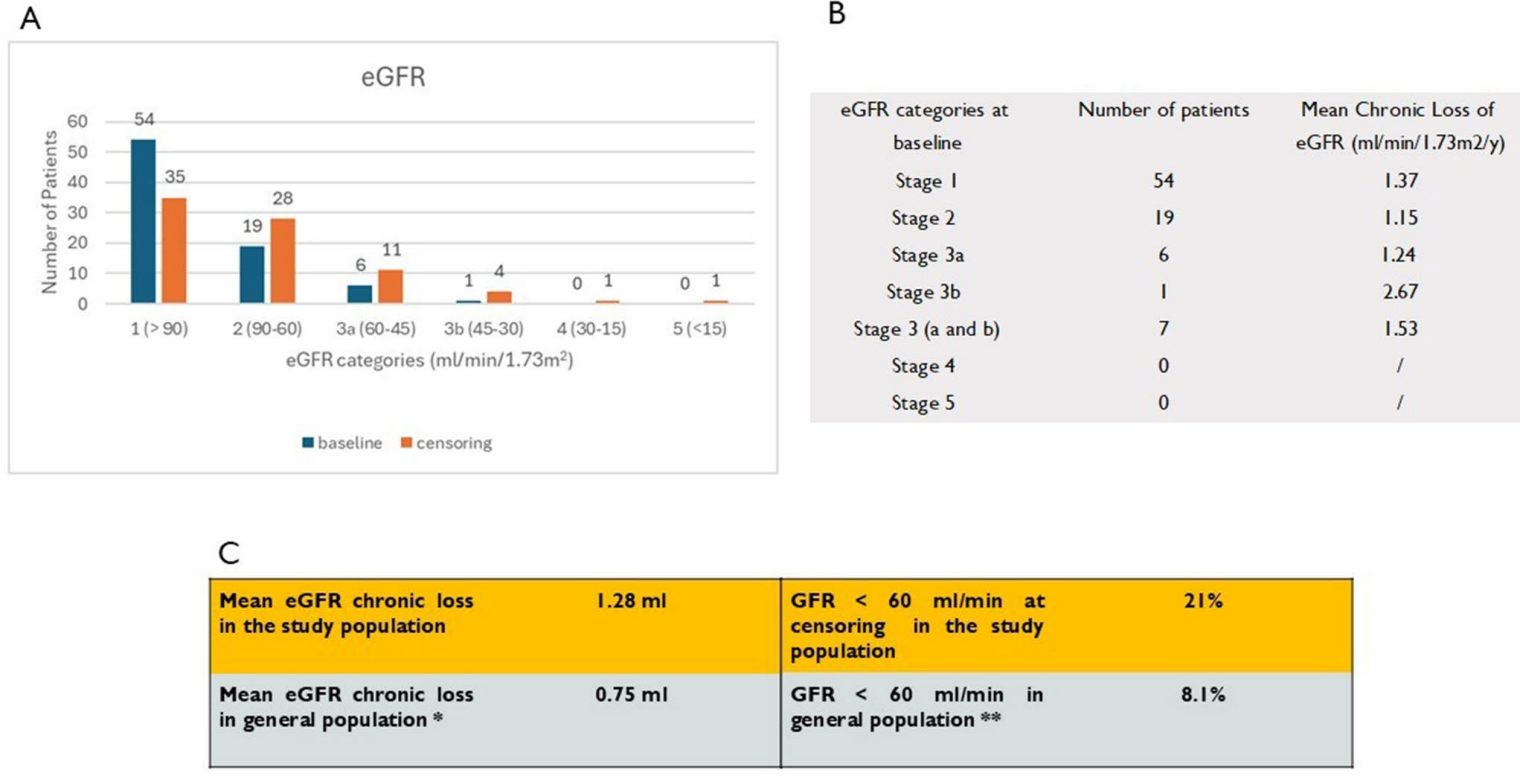
Histogram illustrating the distribution of patients across CKD stages at baseline and at study censoring, depicting the associated chronic loss of eGFR (in mL/min/1.73 m²) for each CKD stage. * Mean chronic loss of our population was compared to a longitudinal study of renal function in healthy subjects [7]. ** Prevalence of CKD in our population was compared to a pooled meta-analysis assessing prevalence of CKD in general population [16]

The mean follow-up duration for chronic slope evaluation was 11.68 (SD 5.5) years. The mean chronic eGFR decline during follow-up was − 1.28 (SD 1.52) mL/min/1.73 m² per year. Of the 80 patients, 7 (9%) had CKD at baseline, while 17 (21%) developed CKD by the study’s conclusion (Fig. 1A).

At the univariable analysis, age (OR 1.12 per year, *p* < 0.01), presence of diabetes (OR 7.02, *p* < 0.01) and of hypertension (OR 5.29, *p* = 0.04) were significantly associated with CKD, whereas no significant differences were found between patients in remission and those with uncontrolled disease. Similarly, no significant association was observed for medical treatment (Supplementary Table 1).

At the multivariable logistic regression (Table 2B) age (OR 1.09 per year, *p* = 0.008) and diabetes (OR 5.66, *p* = 0.011) confirmed as significant predictors of CKD.

## Discussion

Our study confirms that glomerular hyperfiltration caused by excessive GH secretion is acutely reversible with treatment [2] and reveals that this effect is more pronounced in patients undergoing surgery than in those receiving medical therapy. This likely reflects the rapid suppression of GH hypersecretion achieved through surgery, whereas medical therapy requires more time to normalize IGF-1 levels.

In the long-term, patients with acromegaly experience a greater decline in eGFR compared to the healthy population. Previous studies have reported a mean yearly eGFR decline of 0.75 mL/min in a relatively healthy cohort [7], which included patients with diabetes but excluded those with hypertension or renal disease, and of 0.63 mL/min in healthy kidney donors [8]. In contrast, the eGFR decline in our cohort of patients with acromegaly was nearly twice these rates.

By the end of follow-up, 21% of patients had CKD, underscoring acromegaly as a significant risk factor for kidney damage, contributing to greater renal function loss than aging alone. Consistent with our findings, Hong et al. [5] reported an association between acromegaly and an increased risk of ESRD. In our cohort, the prevalence of ESRD was 1.25%, higher than the estimated 0.1% in the general population [9]. However, this finding should be interpreted with caution, as it is based on a single case out of 80 patients.

Acromegaly is associated with comorbidities, particularly diabetes and hypertension, both of which negatively impact renal function. In our cohort, diabetes was significantly associated with a greater chronic decline in eGFR, supporting findings from a large cohort study [5] that identified diabetes as a mediator of renal damage risk in acromegaly. Conversely, no predictive relationship between hypertension and eGFR decline was identified in our cohort. These findings suggest that the combined effects of prolonged GH excess and a higher prevalence of diabetes in patients with acromegaly outweigh the impact of hypertension, which, in contrast, is reported to be the leading cause of ESRD in general population [10]. Together, these factors contribute to an increased risk of adverse cardiovascular events in individuals with acromegaly.

No statistically significant associations were observed between disease status (active vs. remission) and the progression of chronic kidney disease (CKD). Our findings agree with other studies, which also found no differences in disease-related comorbidities prevalence between controlled and uncontrolled patients [11, 12]. We hypothesize that the renal function deterioration in acromegaly may primarily be driven by comorbidities, particularly diabetes, rather than directly correlating with disease control.

We also considered the potential impact of treatment with somatostatin receptor ligands (SRL) but found no association with chronic eGFR changes. In evaluating this finding, it has to be considered the impact of several confounding factors. Somatostatin receptor ligands (SRLs) have demonstrated potential in the treatment of chronic kidney disease by reducing renal hyperfiltration and glomerular hypertension [13]; However, they also elevate the risk of hyperglycemia, particularly with pasireotide [14], which could exacerbate kidney damage. This risk can be managed through careful monitoring of diabetes and timely interventions. Additionally, improvements in acromegaly achieved with these therapies may contribute to better glycemic control [15]. Finally, the cohort receiving SRL comprised patients either ineligible for surgery or with incomplete remission following surgery, which may introduce a selection bias.

Limitations of our study include the availability of preoperative data for less than half of the patients, and the heterogeneity of the study cohort in terms of clinical characteristics and follow-up duration. Furthermore, the albumin/creatinine ratio (ACR) was not consistently measured during the follow-up, limiting our ability to classify the degree of cardiorenal risk more precisely. On the other hand, to the best of our knowledge, our study is the first to analyze the long-term progression of renal function in patients with acromegaly, which represents its main strength.

In conclusion, our study demonstrated that:

- Acute treatment of acromegaly leads to normalization of renal hyperfiltration state associated with the disease, occurring more rapidly in surgically treated patients than in those receiving medical therapy.
- A significant proportion of patients with acromegaly develop CKD, particularly those with diabetes mellitus.

Given the frequent coexistence of hypertrophic cardiomyopathy and elevated cardiovascular risk in acromegaly, our findings emphasize the need for careful monitoring and management of cardiovascular risk factors in these patients. Specifically, our data highlight the importance of monitoring renal function (eGFR and ACR), and considering the early use of renin-angiotensin system (RAS) inhibitors, sodium-glucose cotransporter 2 inhibitors (SGLT2i), and glucagon-like peptide-1 receptor agonists (GLP-1 RAs), particularly in patients with diabetes mellitus, in light of recent data demonstrating their ability to mitigate cardio-renal -metabolic risk.

**Table 1:** Demographic and clinical characteristics of our cohort of patients with acromegaly. Data are expressed as mean (standard deviation) for continuous variables and as frequency (percentage) for categorical variables. BMI, body mass index; eGFR, estimated glomerular filtration rate; DA, dopamine agonist; SRL, somatostatin receptor ligands; GHRA, growth hormone receptor antagonist

Demographic and Clinical Characteristics	Acromegaly cohort (*n* = 80)
Age at diagnosis (years)	43.7 (14.0)
Duration of disease (years)	22.3 (12.7)
Sex (F)	34 (42.5%)
Baseline BMI (kg/m2)	26.9 (4.9)
Baseline eGFR (mL/min/1.73 m2)	99.0 (19.3)
Comorbidities at baseline	
Diabetes	25 (31.3%)
Hypertension	52 (65.0%)
Dislipidemia	29 (36.3%)
Acromegaly treatment	
Surgery	51 (63.8%)
Radiotherapy	11 (13.8%)
Medical Therapy (at last follow-up)	58 (75.0%)
DA	6 (10.3%)
DA + SRL	8 (13.8%)
SRL	34 (58.6%)
GHRA	10 (17.2%)
Remission	55 (68.8%)

**Table 2A:** Acute loss of eGFR. Linear regression analysis examining the acute loss (up to one year after intervention) of estimated eGFR associated with different treatment groups and patients’ characteristics

Acute Loss of eGFR	Coefficient	SE	*p*-value
Therapy (mean baseline eGFR ± SD)			
(i) Medical Therapy (101.3 ml ± 12.3)	Reference		
(ii) Medical Therapy + Surgery* (103.6 ml ± 13.6)	1.16 ml	5.62	0.838
(iii) Only Surgery* (107.9 ml ± 12.3)	-13.32 ml	6.37	0.049
Age (per year)	-0.46 ml	0.22	0.054
Sex (female)	1.45 ml	4.11	0.726
Diabetes	2.13 ml	5.43	0.7
Hypertension	-7.05 ml	4.61	0.14

* Compared to Medical Therapy (reference variable)

**Table 2B:** Risk of CKD. Logistic regression analysis showing the probability of having CKD at last follow-up, based on treatment groups and patients’ characteristics

CKD	OR	SE	*p*-value
Surgery	1.15	0.81	0.837
Age (*per year*)	1.09	0.35	**0.008**
Sex (*female)*	0.77	0.53	0.708
Diabetes	5.66	3.85	**0.011**
Hypertension	1.63	1.49	0.586

SE, Standard Error; OR, Odds Ratio; eGFR, estimated Glomerular Filtration Rate; CKD, Chronic Kidney Disease

## Electronic supplementary material

Below is the link to the electronic supplementary material.

Supplementary Material 1

## Data Availability

No datasets were generated or analysed during the current study.

## References

[1] Kamenicky P, Mazziotti G, Lombes M, Giustina A, Chanson P (2014) Growth hormone, insulin-like growth factor-1, and the kidney: pathophysiological and clinical implications. Endocr Rev 35(2):234–281 24423979 10.1210/er.2013-1071

[2] Fujio S, Takano K, Arimura H, Habu M, Bohara M, Hirano H et al (2016) Treatable glomerular hyperfiltration in patients with active acromegaly. Eur J Endocrinol 175(4):325–333 27440194 10.1530/EJE-16-0242

[3] Kirsch N, Chang LS, Koch S, Glinka A, Dolde C, Colozza G et al (2017) Angiopoietin-like 4 is a Wnt signaling antagonist that promotes LRP6 turnover. Dev Cell 43(1):71–82 e6 29017031 10.1016/j.devcel.2017.09.011

[4] Bostan H, Kizilgul M, Calapkulu M, Kalkisim HK, Topcu FBG, Gul U et al (2024) The prevalence and associated risk factors of detectable renal morphological abnormalities in acromegaly. Pituitary 27(1):44–51 38064149 10.1007/s11102-023-01370-z

[5] Hong S, Kim KS, Han K, Park CY (2023) A cohort study found a high risk of end-stage kidney disease associated with acromegaly. Kidney Int 104(4):820–827 37490954 10.1016/j.kint.2023.06.037

[6] Giustina A, Biermasz N, Casanueva FF, Fleseriu M, Mortini P, Strasburger C et al (2024) Consensus on criteria for acromegaly diagnosis and remission. Pituitary 27(1):7–22 37923946 10.1007/s11102-023-01360-1 PMC10837217

[7] Lindeman RD, Tobin J, Shock NW (1985) Longitudinal studies on the rate of decline in renal function with age. J Am Geriatr Soc 33(4):278–285 3989190 10.1111/j.1532-5415.1985.tb07117.x

[8] Glassock RJ, Denic A, Rule AD (2017) The conundrums of chronic kidney disease and aging. J Nephrol 30(4):477–483 27885585 10.1007/s40620-016-0362-x

[9] Kovesdy CP (2022) Epidemiology of chronic kidney disease: an update 2022. Kidney Int Suppl (2011) 12(1):7–11 10.1016/j.kisu.2021.11.003 PMC9073222 35529086

[10] Kim CS, Kim B, Choi HS, Bae EH, Ma SK, Han KD et al (2021) Cumulative hypertension burden and risk of end-stage renal disease. Hypertens Res 44(12):1652–1661 34408283 10.1038/s41440-021-00723-0

[11] Campana C, Cocchiara F, Corica G, Nista F, Arvigo M, Amaru J et al (2021) Discordant GH and IGF-1 results in treated acromegaly: impact of GH cutoffs and mean values assessment. J Clin Endocrinol Metab 106(3):789–801 33236108 10.1210/clinem/dgaa859

[12] Amodru V, Petrossians P, Colao A, Delemer B, Maione L, Neggers S et al (2020) Discordant biological parameters of remission in acromegaly do not increase the risk of hypertension or diabetes: a study with the Liege acromegaly survey database. Endocrine 70(1):134–142 32562181 10.1007/s12020-020-02387-1

[13] Oh Y (2012) The insulin-like growth factor system in chronic kidney disease: pathophysiology and therapeutic opportunities. Kidney Res Clin Pract 31(1):26–37 26889406 10.1016/j.krcp.2011.12.005 PMC4715090

[14] Gadelha MR, Kasuki L, Lim DST, Fleseriu M (2019) Systemic complications of acromegaly and the impact of the current treatment landscape: an update. Endocr Rev 40(1):268–332 30184064 10.1210/er.2018-00115

[15] Stormann S, Meyhofer SM, Groener JB, Faust J, Schilbach K, Seufert J et al (2024) Management of pasireotide-induced hyperglycemia in patients with acromegaly: An experts’ consensus statement. Front Endocrinol (Lausanne). 2024 Feb 9:15:1348990. 10.3389/fendo.2024.1348990 PMC10884330 38405148

[16] Hill NR, Fatoba ST, Oke JL, Hirst JA, O’Callaghan CA, Lasserson DS et al (2016) Global prevalence of chronic kidney Disease - A systematic review and Meta-Analysis. PLoS ONE 11(7):e0158765 27383068 10.1371/journal.pone.0158765 PMC4934905

