# Supplementary material for: Kidney function in acromegaly: evidence from a long-term observational study
Source: Pituitary. 2025 May 6;28(3):56. doi: 10.1007/s11102-025-01520-5 (PMC12055623; doi:10.1007/s11102-025-01520-5)
Supplement: Supplementary file 1 — Supplementary Material 1 [file 11102_2025_1520_MOESM1_ESM.docx]

**Supplementary Table 1**

| *CKD* | OR | SE | p-value |
| --- | --- | --- | --- |
| Age (per year) | 1.12 | 0.03 | <0.01 |
| Sex (female) | 0.58 | 0.32 | 0.33 |
| Diabetes | 7.02 | 4.23 | <0.01 |
| Hypertension | 5.29 | 4.23 | 0.04 |
| Surgery | 0.43 | 0.24 | 0.14 |
| SRL | 0.91 | 0.51 | 0.87 |
| Remission | 1.26 | 0.75 | 0.70 |

*Supplementary Table Description*

**Supplementary Table 1**

Risk of CKD. Univariate logistic regression analysis assessing the probability of having CKD at last follow-up, according to patients’ demographics, comorbidities, treatment, and disease status.

SE, Standard Error; OR, Odds Ratio; CKD, Chronic Kidney Disease; SRL, Somatostatin Receptor Ligands.
